# Neural correlates of attention‐executive dysfunction in lewy body dementia and Alzheimer's disease

**DOI:** 10.1002/hbm.23100

**Published:** 2015-12-26

**Authors:** Michael Firbank, Xenia Kobeleva, George Cherry, Alison Killen, Peter Gallagher, David J. Burn, Alan J. Thomas, John T. O'Brien, John‐Paul Taylor

**Affiliations:** ^1^ Institute of Neuroscience, Newcastle University, Campus for Ageing and Vitality, Newcastle upon Tyne NE4 5PL United Kingdom; ^2^ Department of Neurology and Neurophysiology Medical School Hannover, Carl‐Neuberg‐Straße 1 Hannover 30625 Germany; ^3^ School of Medical Science Newcastle University, Newcastle‐upon‐Tyne NE2 4HH United Kingdom; ^4^ Department of Psychiatry University of Cambridge School of Clinical Medicine, Cambridge CB2 0SP United Kingdom

**Keywords:** Alzheimer's disease, attention, attention network test, executive, functional MRI, Lewy body dementia

## Abstract

Attentional and executive dysfunction contribute to cognitive impairment in both Lewy body dementia and Alzheimer's disease. Using functional MRI, we examined the neural correlates of three components of attention (alerting, orienting, and executive/conflict function) in 23 patients with Alzheimer's disease, 32 patients with Lewy body dementia (19 with dementia with Lewy bodies and 13 with Parkinson's disease with dementia), and 23 healthy controls using a modified Attention Network Test. Although the functional MRI demonstrated a similar fronto‐parieto‐occipital network activation in all groups, Alzheimer's disease and Lewy body dementia patients had greater activation of this network for incongruent and more difficult trials, which were also accompanied by slower reaction times. There was no recruitment of additional brain regions or, conversely, regional deficits in brain activation. The default mode network, however, displayed diverging activity patterns in the dementia groups. The Alzheimer's disease group had limited task related deactivations of the default mode network, whereas patients with Lewy body dementia showed heightened deactivation to all trials, which might be an attempt to allocate neural resources to impaired attentional networks. We posit that, despite a common endpoint of attention‐executive disturbances in both dementias, the pathophysiological basis of these is very different between these diseases. *Hum Brain Mapp 37:1254–1270, 2016*. © **2015 The Authors Human Brain Mapping Published by Wiley Periodicals, Inc**.

**Abbreviations**
ANT: attention network test
CAMCOG: Cambridge cognitive examination
DMN: default mode network
LBD: Lewy body dementia
MCI: mild cognitive impairment
MMSE: mini‐mental state examination
NPI: neuropsychiatric inventory
ROI: region of interest
RT: reaction times
UPDRS: unified Parkinson's disease rating scale

## INTRODUCTION

Lewy body dementia (LBD) includes both dementia with Lewy bodies and Parkinson's disease dementia, and is a major cause of dementia after Alzheimer's disease, representing 10%–15% of all late onset dementia cases [Vann Jones and O'Brien, 2014]. LBD is characterized by fluctuations in cognition, spontaneous motor features of parkinsonism, complex visual hallucinations as well as a wide array of other symptoms including autonomic dysfunction and sleep disturbances [Emre et al., 2007; McKeith et al., 2005].

Deficits in attention and executive functioning are a common feature across a range of different neurodegenerative dementias [Bosboom et al., 2004; Fernandez‐Duque and Black, 2006; McGuiness et al., 2010], with studies demonstrating that attentional difficulties in dementia with Lewy bodies, and Parkinson's disease with dementia are similar to each other, but more pronounced than in Alzheimer's Disease [Baddeley et al., 2001; Ballard et al., 2002; Ferman et al., 2006; Metzler‐Baddeley, 2007]. Apart from impairing goal‐directed behavior and having profound sequelae for patients and carers in terms of activities of daily living [Bronnick et al., 2006; Lee et al., 2013], attention‐executive deficits and cognitive fluctuations in LBD have also been implicated in the aetiology of hallucinations [Meppelink et al., 2008; Shine et al., 2011] and so have a more diverse and larger impact on patients. However, the origin of attention‐executive deficits in LBD and, in particular, which neuroanatomical substrates of attentional dysfunction distinguish LBD from Alzheimer's disease remains unclear.

Posner and colleagues [Fan et al., 2002; Posner and Petersen, 1990] suggested that attention can be modelled as having three functionally inter‐related but anatomically distinct components: alerting, a function which relates to achieving and maintaining an alert state; orienting, which allows the selection of information from sensory input, and executive control (conflict resolution). Alerting may be dependent upon the brain stem and its connectivity with the frontoparietal cortex [Rinne et al., 2013], whereas the orienting function appears to be dependent upon activity in aspects of the dorsal attentional network, including the superior parietal lobule and frontal eye fields, and regions of the ventral attentional network such as the temporoparietal junction and inferior frontal gyrus [Corbetta and Shulman, 2002; Kincade et al., 2005]. Executive function has been mostly related to frontal executive control networks [Dosenbach et al., 2006]. On the other hand, opposing these task‐positive networks, there is a general task negative network, the default mode network (DMN), which includes the midline and inferior parietal cortex [Binder, 2012; Binder et al., 1999; Greicius et al., 2003; Shulman et al., 1997]. Successful task performance appears contingent upon the allocation of neural resources to the task‐positive regions, which is mediated by deactivation of the DMN [Raichle et al., 2001; Sidlauskaite et al., 2014; Singh and Fawcett, 2008].

The three domains of attention described by Posner and colleagues can be delineated by the attention network test (ANT) [Fan et al., 2002]. This task has been successfully used, behaviourally, in people with dementia with Lewy bodies [Fuentes et al., 2010] and Alzheimer's disease [Fernandez‐Duque and Black, 2006] and it avoids performance confounder effects by achieving reasonable accuracy and compliance in all participants whilst still engaging the control participants. However whilst it has been applied in mild cognitive impairment [Van Dam et al., 2013] and non‐demented Parkinson's disease [Madhyastha et al., 2015] during functional neuroimaging, the ANT has not, to date, been used to examine neural correlates of attention in different dementia groups.

Our study aim, therefore, was to investigate the neural correlates of different subcomponents of attentional function using a version of the ANT in LBD and Alzheimer's disease in comparison to each other and a healthy aged control group using functional magnetic resonance imaging (fMRI). We attempted to identify how attention brain regions were altered in people with dementia, and to determine to what extent this varied between these dementia subtypes. In particular, given the extensive literature associating dysfunction of the DMN with neurodegeneration [Buckner et al., 2008; Hafkemeijer et al., 2012] we also focussed on the role of the DMN and its deactivation in the executive component of the task, how this differed with dementia type, and, how it was modulated by task difficulty.

We hypothesized that we would find less task related deactivation of the DMN in Alzheimer's disease as demonstrated by previous studies [Rombouts et al., 2005; Sperling, 2011], and also possibly in LBD given the previous varied reports [Franciotti et al., 2013; Sauer et al., 2006]. We also hypothesized that we would find frontal impairment in Alzheimer's disease and, that in Lewy body dementia we would see both frontal and posterior cortical dysfunction, in line with experimental findings from previous neuroimaging studies [Binnewijzend et al., 2014; Mak et al., 2015] that support the dual‐syndrome hypothesis, [Kehagia, 2013] which suggests that cognitive impairment in PD is a combination of (a) dopaminergic executive fronto‐striatal dysfunction and (b) cholinergic related visuospatial posterior cortical and temporal lobe dysfunction. From a behavioural perspective, in accordance with previous behavioural studies, we expected to see slowed executive processing in both dementias [Fernandez‐Duque and Black, 2006; Wang et al., 2013; Wylie et al., 2007].

## MATERIALS AND METHODS

### Participants

Study participants were recruited between September 2010 and January 2014 prospectively from people aged over 60 with mild to moderate dementia with a Mini‐Mental State Examination (MMSE) score >12 from a local community‐dwelling population of participants who had been referred to local old age psychiatry and neurology services. Healthy controls were selected from friends and spouses of participants included in this and previous studies. The study was approved by the local ethics committee.

Diagnosis of probable dementia with Lewy bodies, Parkinson's disease with dementia or Alzheimer's disease was made independently by two experienced clinicians using the revised International Consensus Guidelines for dementia with Lewy bodies [McKeith et al., 2005], diagnostic criteria for PDD [Emre et al., 2007] and the National Institute on Aging‐Alzheimer's Association (NIA‐AA) criteria for Alzheimer's disease [McKhann et al., 2011], respectively. Cognitive function was tested using the Cambridge Cognitive Examination (CAMCOG, maximum score 105) and the MMSE (maximum score 30). The presence and severity of any extrapyramidal signs were graded using the motor component of the Unified Parkinson's disease rating scale (UPDRS). Cognitive fluctuations were assessed using the MAYO scale [Ferman et al., 2004], clinical assessment of fluctuations [Walker et al., 2000] and the Neuropsychiatric Inventory was also administered [Cummings et al., 1994]. Depressive features were assessed with the Cornell scale for depression in dementia [Alexopoulos et al., 1988], executive function was measured using phonemic fluency (words beginning with F,A,S in one minute each) [Benton, 1968]. Visuospatial function was assessed with an angle discrimination task [Mosimann et al., 2004] in which subjects were required to match the angle of a single line to one of five lines forming a semicircle.

Control participants in the study demonstrated no evidence of dementia (from history and score >80 on CAMCOG). Exclusion criteria for all participants included contra‐indications for MR imaging, moderate to severe visual impairment, previous history of alcohol or substance misuse, significant neurological or psychiatric history, moderate to severe cerebral small vessel disease, focal brain lesions on brain imaging or the presence of other severe or unstable medical illness.

Before undergoing a scanning session and formal in‐scan testing with the ANT, participants were familiarized with the task, and it was verified that they could perform it correctly (task accuracy > 70%). All LBD patients were scanned whilst taking their usual anti‐parkinsonian medications and in an “on” motor state.

### Task

**Figure 1:**
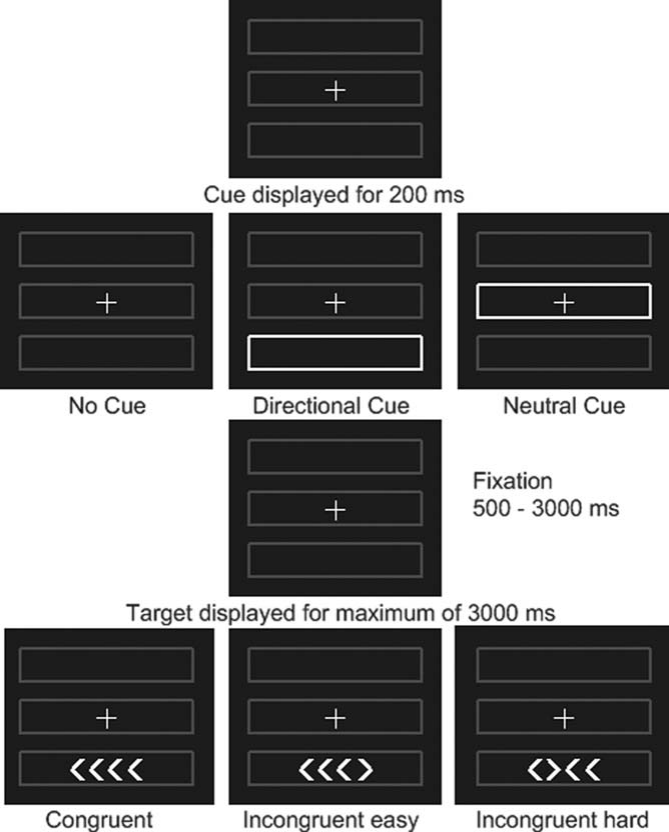
Task design for ANT task. Each trial was either initiated by no cue, a neutral or directional cue, followed by a target after a fixation period of variable length. The target was either congruent (all arrows pointing into one direction) or incongruent (one arrow pointing into the opposite direction). The participants were instructed to push a button depending on the direction of the majority of the arrows.

The task was based on the ANT [Fan et al., 2002] with a modified target component. In the original ANT, participants had to indicate the direction of an arrow which is surrounded by flankers which are either the same or different. In our version, we incorporated a graded conflict task to examine any potential executive dysfunction in our dementia groups in greater depth; participants were shown four arrowheads (horizontal spacing between arrows 0.48 degrees), and had to indicate the direction of the majority (Fig. 1). The four arrowheads were either all pointing the same direction (congruent), or one of the arrows pointing the opposite direction (incongruent). The incongruent arrow appeared either on the end of the row (easy incongruent) or as one of the middle two (hard incongruent). Hence, the easy incongruent task had three congruent arrows in a row (unilateral flanker effect), whereas the hard task had only two (bilateral flanker effect), and therefore provided greater conflict, and a longer reaction time. Behavioral contrasts were defined as (a) alerting effect = mean RT of the no cue trials minus neutral cue trials; (b) orienting effect = mean RT of neutral cue trials minus directional cue trials; (c) Executive effect = mean RT of all (easy and hard) incongruent trials minus congruent trials; (d) Conflict effect = mean RT of the hard incongruent minus easy incongruent trials.

### fMRI Stimulus Presentation

Visual stimuli were back‐projected on to a screen at the foot of the scanner, and participants viewed the stimuli via a mirror positioned above their eyes. All participants had measurement of their best near visual acuity on Landolt broken rings and fMRI compatible goggles with lenses that ranged from −4.0 to 4.0 diopters (0.5 increment) were used to correct any refractive errors that participants had.

Participants looked at a screen with a central cross hair, and three grey boxes (Fig. 1). For each run there were 36 trials. On each trial, a cue was presented for 200 ms—either no cue (no change), neutral cue (in which the central box lit up), or a directional cue (the box in which the target would appear lit up). All directional cues were valid. For the target, four arrow heads appeared in either the upper or lower box. These were either all pointing the same direction (congruent), or one arrow pointing the opposite direction (incongruent). The target remained on screen until a response was made, or 3000 ms had elapsed. The time between the disappearance of the cue and the onset of the target was exponentially distributed at times of 700, 770, 850, 960, 1080, 1240, 1430, 1660, 1940, 2300, 2700, 3200 ms, and the time between the onset of the target, and the onset of the next cue was one of 4300, 4500, 4750, 5000, 5350, 5700, 6100, 6400, 6800, 7200, 7700, 8300 ms with each duration occurring three times in random order during a run. Each cue appeared 12 times, and there were 18 congruent trials and 18 incongruent trials (equally split in easy and hard) per run. The stimulus was programmed in Matlab (Mathworks, Natick, Massachusetts) using the cogent toolbox (http://www.vislab.ucl.ac.uk/cogent_2000.php)

### Neuroimaging Data Acquisition

Participants were scanned on a 3T whole body MR scanner (Achieva scanner; Philips Medical System, the Netherlands), with body coil transmission and eight channel head coil receiver. Images acquired included a standard whole brain structural scan (3D MPRAGE, sagittal acquisition, slice thickness 1.0 mm, in plane resolution 1.0 × 1.0 mm^2^; TR = 8.3 ms; TE= 4.6 ms; flip angle = 8°; SENSE factor = 2). fMRI data were collected with a gradient‐echo (GE) echo planar imaging (EPI) sequence (TR = 1.92 s; TE = 40 ms; field of view (FOV) 192 × 192 mm^2^ 64 × 64 matrix size, flip angle 90°, 27 slices, slice thickness 3 mm, slice gap 1 mm) with 156 volumes (five minutes). We collected between four and six runs of fMRI data whilst participants performed the attention task. We excluded those runs with < 2/3 correct responses as performance per run worse than this was not significantly different from chance.

### fMRI Analysis

We used SPM8 (http://www.fil.ion.ucl.ac.uk/spm/) for all image analysis. For each participant, the T1 anatomical image was segmented and spatially normalised in SPM using the default parameters. We then used the DARTEL [Ashburner, 2007] toolbox to refine the spatial normalisation and create a custom template. The fMRI data were first motion corrected by aligning all functional images to the first image for the subject, and subsequently the mean image. Runs with > 3mm or > 3 degrees head motion were excluded. They were then coregistered with the subject's T1 anatomical image. The spatial normalization parameters from the T1 image were used to write out the EPI data in standard space with a voxel size of 3 × 3 × 3 mm^3^. The normalized images were then smoothed with a 8 × 8 × 8 mm^3^ FWHM (full width half maximum) Gaussian kernel. A high pass filter of 128 seconds was used, and serial correlations were removed with SPM's AR(1) model.

The general linear model (GLM) in SPM was used to conduct a whole‐brain analysis of the fMRI data. We created a design matrix using an impulse function with onset time of the events (separate events for no, neutral and directional cues, and congruent, incongruent‐easy and incongruent‐hard targets with correct responses). Missed targets and incorrectly responded to targets were combined as an extra column in the design matrix. These events were convolved with the canonical haemodynamic response function (HRF), and the first derivative of the HRF was also included to model variation in onset latency.

The six parameters from the motion correction for each functional run were included in the design matrix as covariates of no interest. The regressors were fitted to the fMRI data to produce beta estimates for each regressor. Individual subject and second level (random effects) group analyses were conducted. Contrasts were as the behavioral analysis, except inverted (ie alerting fMRI effect = neutral cue beta estimate – no cue). Only effects surviving an uncorrected voxelwise threshold of *P* < 0.001 and a clusterwise familywise error (FWE) corrected threshold of *P* < 0.05 were interpreted.

Regions of interest (ROIs) were defined from the incongruent vs congruent contrast over all participants. The whole group incongruent > congruent voxelwise statistics were thresholded at FWE *P* <0.05, and the voxels surviving this threshold were manually divided into distinct anatomical regions to define the activation ROIs (Supporting Information Fig. S1). For the DMN, we thresholded the congruent > incongruent voxelwise analysis at *P* < 0.001 uncorrected, and created two ROIs (frontal and parietal) from the thresholded voxels. We investigated deactivations in the DMN by examination of the blood‐oxygen‐level dependent **(**BOLD) signal during the targets compared to baseline in all participants within the two DMN ROIs (frontal and parietal) which are integral to this network.

In order to investigate the magnitude of the BOLD signal during task related deactivations, we utilised the MarsBaR SPM toolbox (http://marsbar.sourceforge.net/) to extract mean values for the BOLD contrast for the comparisons.

## RESULTS

### Demographics

There were 23 controls, 30 Alzheimer's disease, 24 dementia with Lewy bodies, and 22 Parkinson's disease with dementia subjects who completed the protocol. Of these, data were lost because of technical failure with the response device (one Alzheimer's disease, three Parkinson's disease with dementia) excessive motion on MRI (three Alzheimer's disease, three Parkinson's disease with dementia), scanning stopped before sufficient functional scans completed (three Alzheimer's disease, one dementia with Lewy bodies, two Parkinson's disease with dementia), and insufficiently accurate task performance (four dementia with Lewy bodies, one Parkinson's disease with dementia). This left 23 controls (21 with six runs and 2 with five runs of fMRI data), 23 Alzheimer's disease (15 with six runs, 1 with five runs, and 7 with four runs of fMRI data), 19 dementia with Lewy bodies (12 with six runs, 4 with five runs, and 3 with four runs of fMRI Data), and 13 Parkinson's disease with dementia (12 with six runs, 1 with five runs).

Those with Parkinson's disease and dementia had a significantly higher UPDRS motor score, were on higher l‐Dopa equivalent doses (all of the Parkinson's disease with dementia patients were taking l‐dopa compared with 8/19 (42%) of the dementia with Lewy bodies patients), and had higher scores for the Cornell depression scale, the total neuropsychiatric inventory, and the MAYO and CAF than dementia with Lewy bodies patients (Supporting Information Table S1). However, there were no significant differences between these two groups in cognitive performance on the MMSE, CAMCOG, FAS, or visuospatial tests.

Since previous studies have found a similar profile of attentional and executive function between dementia with Lewy bodies, and Parkinson's disease with dementia patients [Ballard et al., 2002], we decided apriori to combine these patients together. To verify that performance on the ANT was indeed similar between the two groups, we compared behavioral and imaging performance. Supporting Information Table S2 shows the behavioral results and Supporting Information Table S3 the imaging results. There were no significant RT differences, and only one significant difference (*P* = 0.03) over all ROIs and target/cue comparisons. Results from the combined dementia with Lewy bodies and Parkinson's disease with dementia patients group are presented in the rest of the paper as a single LBD group (*n* = 32).

Table 1 presents demographic data on those with successful MRI data. Compared to Alzheimer's disease, the LBD group, as expected, had higher UPDRS motor score, worse performance on the angle discrimination task, and higher score on the MAYO fluctuation score, although there were no differences in CAMCOG global or executive function between Alzheimer's disease and LBD.

**Table 1:** Demographics and clinical scores

	Controls (*N* = 23)	Alzheimer's disease (*N* = 23)	LBD (*N* = 32)	Between group differences (*P*‐value)	Between group post‐hoc tests
Age	76.3 (5.5)	75.8 (8.2)	75.0 (6.4)	*F*_2,75_ = 0.25,*P* = 0.8	–
Sex male:female	16:7	20:4	27:5	*χ* ^2^ = 2.0; *P* = 0.4	–
Duration (years) cognitive decline	–	3.39 (1.61)	3.33 (2.07)	*T* = 1.2; *P* = 0.9^a^	–
Cholinesterase Inhibitors	0 (0%)	25 (100%)	27 (84%)	Fisher p =0.068^a^	–
Positive DAT scan	–	–	10/11 (91%)		–
UPDRS	1.3 (1.7)	2.0 (1.7)	19.06 (8.0)	*F*_2,75_ = 102, *P* < 0.001	Con = AD « LBD
Cornell	0.59 (1.1)	0.91 (1.1)	2.97 (2.2)	*F*_2,75_ = 18, *P* < 0.001	Con = AD « LBD
MMSE	29.1 (0.9)	22.0 (3.2)	23.4 (3.8)	*T* = 1.4; *P* = 0.16^a^	–
CAMCOG	96.8 (3.6)	71.0 (11.7)	76.7 (12.6)	*T* = 1.7; *P* = 0.10^a^	–
CAMCOG executive	23.0 (2.5)	15.1 (4.1)	13.3 (4.1)	*T* = 1.7; *P* = 0.10^a^	–
MAYO fluctuations		1.0 (1.0)	2.35 (1.4)	*T* = 3.9;*P* < 0.001^a^	–
MAYO cognitive		1.95 (1.9)	2.71 (1.9)	*T* = 1.4; *P* = 0.16^a^	–
CAF total	–	0.5 (1.4)	4.81 (4.1)	*T* = 4.8; *P* < 0.001	–
1 day fluctuation		2.14 (2.4)	3.59 (3.1)	*T* = 1.8; *P* = 0.08^a^	–
NPI		6.2 (6.8)	13.4 (10.0)	*T* = 3.0; *P* = 0.005^a^	–
Verbal fluency (FAS)	43.7 (16.2)	30.1 (15.5)	20.5 (11.9)	*F*_2,75_ = 17,*P* < 0.001	Con » AD › LBD
Angle discrimination	19.7 (0.8)	19.4 (1.3)	16.5 (4.7)	*F*_2,75_ = 8.7, *P* < 0.001	Con = AD » LBD

a Alzheimer's disease vs Lewy body dementia 2 group comparison.

ANOVA Post hoc (Tukey) group comparisons, AD, Alzheimer's disease; Con, controls.

‹ Indicates *P* < 0.05; « *P* < 0.01; = indicates *P* >=0.05.

Abbreviations: CAMCOG, Cambridge Cognitive Examination; MMSE, Mini ‐Mental State Examination; NPI, Neuropsychiatric Inventory; CAF, Clinician Assessment of Fluctuations Scale; UPDRS, Unified Parkinson's disease rating scale; FAS, fluency for words starting with F,A,& S; Cornell, Cornell depression in dementia rating scale.

### Behavioral Task Data

The minimum number of trials responded to was 74%, with a minimum of 71% of all trials being correctly responded to. Table 2 presents the error rates and reaction times to trials with correct responses in the scanner from those runs included in the fMRI analysis. Controls responded to more trials, and had more correct responses than the dementia groups, but there were no significant differences in error rate between Alzheimer's disease and LBD groups. Whilst the error rate in controls did not differ between conditions, we found a significant increase in error rate in both LBD and Alzheimer's disease patients when comparing the incongruent and congruent condition.

**Table 2:** Reaction times and accuracy

	Controls (*n* = 23)	Alzheimer's disease (*n* = 23)	LBD (*n* = 32)	Between group Anova (df = 2,75) comparison (*P*‐value)	Tukey Post hoc between group comparisons
ANT trials responded % [range]	99.6 (0.89) [96.8 – 100]	95.9 (6.6) [74.5–100]	96.7 (4.72) [78.7–100]	*P* = 0.024	Con › AD =LBD
**RT for correct trials (ms)**					
No cue	928 (177)	1,152 (247)	1,395 (300)	*P* < 0.001	Con ‹ AD « LBD
Neutral cue	896 (159)	1,133 (261)	1,391 (322)	*P* < 0.001	Con « AD « LBD
Directional cue	816 (178)	1,060 (241)	1,325 (330)	*P* < 0.001	Con « AD « LBD
Congruent	714 (121)	897 (210)	1,125 (262)	*P* < 0.001	Con ‹ AD « LBD
All Incongruent	1,044 (233)	1,347 (312)	1,652 (410)	*P* < 0.001	Con « AD « LBD
Incongruent easy	1,005 (217)	1,229 (274)	1,521 (391)	*P* < 0.001	Con ‹ AD « LBD
Incongruent hard	1,084 (265)	1,477 (384)	1,800 (486)	*P* < 0.001	Con « AD ‹ LBD
**Correct responses (%)**					
No cue	98.4 (1.9)	91.0 (8.3)	89.2 (7.6)	*P* < 0.001	Con » AD = LBD
Neutral cue	98.6 (1.3)	90.7 (9.2)	87.7 (9.3)	*P* < 0.001	Con » AD = LBD
Directional cue	98.9 (1.5)	91.8 (9.2)	88.1 (8.4)	*P* < 0.001	Con » AD = LBD
Congruent	98.8 (1.3)	94.9 (6.3)	94.0 (6.3)	*P* = 0.004	Con › AD; Con » LBD; AD = LBD
All incongruent	98.4 (1.4)	87.4 (12.1)	82.7 (12.3)	*P* < 0.001	Con » AD = LDB
Incongruent easy	98.2 (2.3)	88.2 (13.1)	84.6 (13.3)	*P* < 0.001	Con » AD = LDB
Incongruent hard	98.6 (1.5)	86.7 (13.5)	80.9 (14.5)	*P* < 0.001	Con » AD = LDB
**RT differences (ms)**					
Alerting	32.1 (48)*	19.8 (93)^†^	4.7 (88)^†^	0.5	–
Orienting	79.9 (55)**	72.7 (83)**	66.1 (95)**	0.8	–
Executive	330 (151)**	451 (191)**	527 (203)**	0.001	LBD « Con; AD=Con; AD=LBD
Conflict	79 (129)*	248 (228)**	279 (249)**	0.003	Con « LBD; Con « AD; AD=LBD
**Error rate differences (%)**					
Alerting	−0.17 (1.85)	0.31 (6.35)	1.48 (4.36)	0.383	–
Orienting	0.25 (1.38)	1.07 (3.44)	0.39 (3.96)	0.648	–
Executive	0.39 (1.25)	7.50 (9.61)**	11.24 (10.84)**	<0.001	Con « LBD; Con « AD; AD=LBD
Conflict	−0.40 (2.73)	1.46 (10.98)	3.71 (13.02)	0.347	–

Group means for reaction times (RT) and accuracy within and between different conditions (alerting = no cue – neutral cue, orienting = neutral cue – directional cue, executive = incongruent – congruent, conflict = hard — easy). The standard deviations are presented in brackets.

Within group one sample *t* test for difference between cues/targets:.

*P < 0.05.

**P < 0.001.

^†^Not significant (*P* > 0.15).

For the Tukey Post hoc comparisons in RT, AD, Alzheimer's disease; Con, controls.

‹ indicates *P* < 0.05; « *P* < 0.01; = indicates *P* >=0.0.5

Responses were fastest in controls, then Alzheimer's disease, and slowest in LBD across all cue and target types (Table 2). In the control group, there was an alerting effect (neutral—no cue RT = 32 ms, *P* =0.004), which was not significant in either dementia group, though there were no significant group differences in alerting. There was an orienting effect of similar magnitude in all groups, with responses following the directional cue being significantly faster than to the neutral cue. There was also evidence of an executive‐conflict effect, with congruent RT < easy incongruent RT < hard incongruent RT in all groups, though the difference between the easy and hard incongruent conditions was smaller for the controls than the dementia patients. Although the LBD were overall slower than the Alzheimer's disease patients, there were no significant differences between the dementia groups in alerting, orienting or executive‐conflict behavioural effects.

### Regional fMRI Activity

#### Alerting and orienting activations

**Figure 2:**
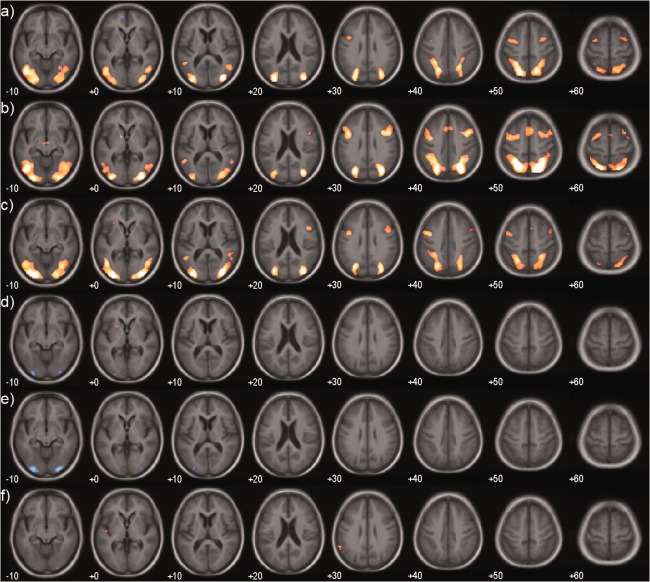
Alerting and orienting effects. Group maps of fMRI activation for alerting (neutral – no cue) for (**a**) Controls, (**b**) Alzheimer's disease, (**c**) LBD (dementia with Lewy bodies+Parkinson's disease with dementia) and for orienting (directional – neutral cue) for (**d**) Controls, (**e**) Alzheimer's disease, (**f**) LBD. Significantly activated voxels (*P* < 0.001 uncorrected) are overlaid on an age matched template in MNI space. Colour overlay is T statistic from −6 (blue) to +6 (yellow). [Color figure can be viewed in the online issue, which is available at http://wileyonlinelibrary.com.]

Figure 2 shows the alerting (neutral cue—no cue) fMRI contrast. There were no significant differences (no significant clusters after voxelwise threshold of *P* = 0.001 uncorrected) between any groups. For the orienting (directional—neutral cue) we did not see any significant increases in activation in any group. However, there was a small bilateral medial occipital region in all groups where activity was greater for neutral vs directional cues, although this cluster was only significant in the Alzheimer's disease group. There were no significant differences between any groups.

We used the frontal and parietal DMN ROIs to investigate deactivation following the cues relative to no cue. The control group had significant parietal deactivation to both cues (neutral cue, *P* = 0.016; directional cue, *P* = 0.025; Supporting Information Fig. S2), and frontal deactivation to neutral cue (*P* = 0.009), whilst LBD had only frontal deactivation to the neutral cue (*P* = 0.021). The Alzheimer's disease group showed a degree of deactivation, but this was not significant for any cue.

#### Executive‐conflict activations

**Figure 3:**
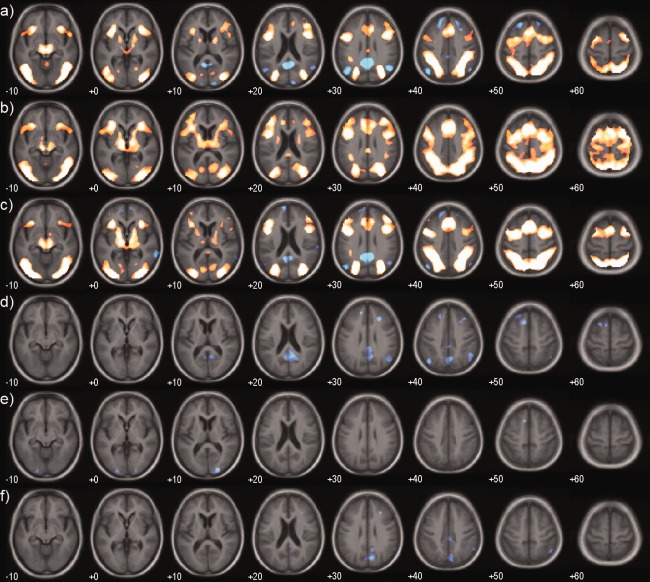
Executive effect. Mean group activations and group contrasts during the contrast incongruent – congruent target. (**a**) Controls, (**b**) Alzheimer's disease, (**c**) LBD, (**d**) Control – AD, (**e**) Control – LBD, (**f**) LBD – AD. Significantly activated voxels (*P* < 0.001 uncorrected) are overlaid on an age matched template in MNI space. Colour overlay is T statistic from −6 (blue) to +6 (yellow). [Color figure can be viewed in the online issue, which is available at http://wileyonlinelibrary.com.]

Figure 3 shows the contrast between the incongruent and congruent targets. There was a fronto–parietal network of activity, along with lateral occipital activation in all groups. The occipital activation was greater in LBD vs controls. Both the control and LBD group demonstrated a significant deactivation in regions associated with the default mode network (DMN). This deactivation was not seen in the Alzheimer's disease group, and there were significant clusters where the deactivation was greater in controls and LBD as compared to Alzheimer's disease.

**Figure 4:**
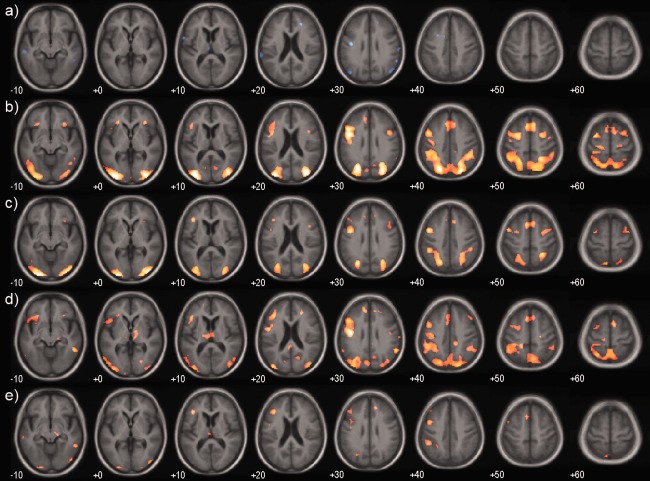
Conflict effect. Mean group activations and group contrasts during the contrast hard – easy congruent target. (**a**) Controls, (**b**) Alzheimer's disease, (**c**) LBD, (**d**) AD – Control, (**e**) LBD – Control. Significantly activated voxels (*P* < 0.001 uncorrected) are overlaid on an age matched template in MNI space. Colour overlay is T statistic from −6 (blue) to +6 (yellow). [Color figure can be viewed in the online issue, which is available at http://wileyonlinelibrary.com.]

Investigating the effect of task difficulty, by comparing the easy vs hard incongruent targets, there was significantly greater occipito‐parietal and frontal activation in the Alzheimer's disease, and to a lesser extent the LBD group, compared with the controls (Fig. 4). In the whole brain analysis, however, there were no differential activity changes between Alzheimer's disease and LBD with increasing task difficulty.

### Region of Interest Analyses

**Figure 5:**
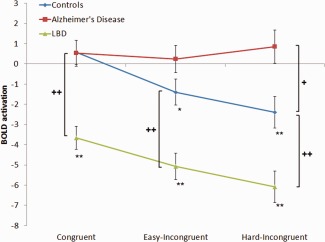
BOLD contrast for the parietal DMN region during target presentation. Parietal DMN ROI analysis showing the BOLD contrast for the parietal DMN region for each group. Error bars are SE. Asterisk indicates within group contrasts (*P<0.05, **P<0.01). Cross indicates between group contrasts (^+^ *P* < 0.05; ^++^ *P* < 0.01). [Color figure can be viewed in the online issue, which is available at http://wileyonlinelibrary.com.]

Supporting Information Tables S4 and S5 present the ROI BOLD contrast data for the targets. Figure 5 shows the BOLD signal for the posterior DMN related ROI for each of the targets in the three groups; no deactivation for the congruent targets but significant deactivation for the two incongruent targets was seen in the control group whereas the LBD group showed deactivation for all targets, with the level increasing with task difficulty. The Alzheimer's disease group did not show deactivation for any of the target types. The frontal DMN demonstrated a similar deactivation pattern across groups in response to the targets.

### Cue × Target Interaction

**Figure 6:**
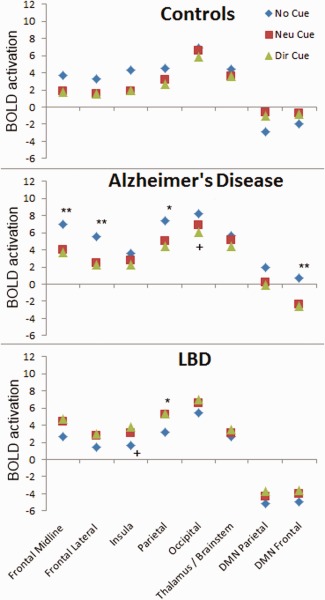
Target activations following different cue conditions. Group activations in the ROIs to targets following different cues (no cue, neutral cue, directional cue). Significant within‐group differences for no‐cue versus neutral cue are marked by an asterix (**P < 0.01 and *P < 0.05) and for neutral versus directional cue by a cross (^+^ *P* < 0.05). [Color figure can be viewed in the online issue, which is available at http://wileyonlinelibrary.com.]

We examined the interaction between the cue and the target using ROI analysis with the regions shown in Supporting Information Figure S1. Figure 6 and Supporting Information Table S6 show the BOLD activations to targets following the different cues. For the controls and Alzheimer's disease groups, there was a tendency (significant in the Alzheimer's disease group) for the no‐cue condition to be followed by greater fronto‐parietal activation to the target. In contrast, the LBD group showed the opposite tendency, with signficantly greater target activation following the cues compared to no‐cue particularly in the parietal ROI. Presentation of a directional cue also led to significantly more activation of the insula during target presentation in LBD.

For the DMN regions, the controls demonstrated a significant target deactivation following the no‐cue condition (parietal DMN, *P* = 0.03), but not to the neutral or directional cues (Supporting Information Table S6). The LBD group, in contrast, had strong DMN deactivation (frontal and parietal) to the target following both cues and no‐cues and the Alzheimer's disease group demonstrated deactivation following the cues only in the frontal DMN (neutral cue, *P* = 0.02; directional cue, *P* = 0.005)

### Invalid Responses

We also investigated the fMRI activation to the targets with invalid (wrong or missed) response vs those with a correct response. For this analysis, we included those participants with at least 15 invalid responses (over all runs); there were no controls, 10 Alzheimer's disease and 21 LBD in this analysis. Supporting Information Table S7 shows the BOLD response for the invalid vs valid responses. For both Alzheimer's disease and LBD, there is more activity for invalid responses, in most regions of the task positive network, and deactivation for the LBD group in the DMN. There were no significant difference between Alzheimer's disease and LBD groups in any region.

## DISCUSSION

In the present study we sought to compare the alerting, orienting and executive networks of attention [Fan et al., 2002] in Alzheimer's disease and LBD by use of a modified ANT. The task was successfully completed by the majority of patients, indicating that it is suitable for investigating attention in participants with mild to moderate dementia. The modification of the ANT task into two grades of conflict also allowed us to study the effect of task difficulty whilst maintaining a reasonable error rate in patients with dementia and thus minimising the confound of poor performance in our dementia groups on functional activity. We found no behavioral and only subtle neuroimaging differences between dementia with Lewy bodies and Parkinson's disease with dementia thus suggesting similar mechanistic processes are engaged during attentional‐executive processing in these groups. This concurs with the significant body of research supporting commonalities in behavior and cognitive deficits between these diseases [Ballard et al., 2002; Noe et al., 2004; Tsuboi et al., 2007].

### Effect of Cue

We investigated the effect of cueing in two ways. Firstly, we examined how brain activation changed directly on presentation of a cue vs no cue. Secondly, we examined the effect of the cue on target response from an fMRI BOLD perspective during target presentation in response to each cue type given evidences supporting cue‐target interactions in the ANT [MacLeod et al., 2010; Weinbach and Henik, 2012] [Galvoa‐Carmona et al., 2014].

### Alerting Effect

We found a strong behavioral alerting effect in controls, but no effect of alerting in either dementia group, which is in line with previous studies showing reduced alerting effects in dementia. Possibly the lack of alerting is because of impaired interactions of the noradrenergic system in the brainstem with frontoparietal regions [Fernandez‐Duque and Black, 2006; Festa‐Martino, 2004; Fuentes et al., 2010; Tales et al., 2005]. When contrasting neutral cue vs no cue on fMRI we found a comparable activation in the fronto‐parietal‐occipital regions in all groups, similar to previous neuroimaging studies which have utilized the ANT [Fan et al., 2005; Liu et al., 2013; Madhyastha et al., 2015; Zheng et al., 2012].

However, the brain activation during target processing was differentially influenced by the alerting effect in all three groups (Fig. 6). In controls, during target presentation, there was a tendency for a decreased frontoparietal activation after the neutral cue (and also spatial cue – see below) compared with no cue and a significant deactivation of DMN when there was no prior cue. This suggests that in the controls the presence of a cue allowed for increased preparation as evidenced by the early deactivation of the DMN during cue (Supporting Information Fig. S2) and less need for dynamical switching between DMN and task postive networks during target presentation and this is further supported by the behavioural benefits of the cues observed in this group.

In Alzheimer's disease, presentation of the neutral cue compared with no cue led to decreased frontoparietal activation as well as deactivation of only the anterior DMN during target presentation. Many studies have found reduced task related deactivation in the DMN in Alzheimer's disease [Celone et al., 2006; Lustig et al., 2003; Rombouts et al., 2009]. However although the presence of a cue may initiate neural switching from the DMN to the task positive networks prior to the onset of the target [Pihlajamaki and Sperling, 2009; Sidlauskaite et al., 2014], the consequent decreased frontoparietal activation, if reflecting a degree of preparation for the target presentation, does not appear to translate into any significant behavioural benefits for the Alzheimer's disease group. One explanation is that the partial DMN deactivation evidenced in this group, and in particular the failure to deactivate the posterior DMN, undermined the efficient allocation of attention during task [Leech et al., 2011].

In LBD patients, in contrast to Alzheimer's disease patients and controls, target activations following the cues tended to be higher than following no cue, and this was significantly so in the parietal region. This enhanced activation may be a compensatory attempt, as observed in other studies in related conditions such as Parkinson's disease [Helmich et al., 2007], but one which fails due to impaired stimulus processing which typifies LBD patients [Calderon et al., 2001; Mosimann et al., 2004] and difficulty in disengaging from the cue to attend to the task [Thiel et al., 2004].

### Orienting Effect

Both dementia groups and controls benefited from the orienting effect in terms of task speed. In Alzheimer's disease, these findings are supported by a number of behavioral studies showing normal [Festa‐Martino, 2004] or enhanced orienting [Tales et al., 2002], although other researchers found a reduced orienting effect [Fernández et al., 2011]. The intact orienting RT effect in LBD is in contrast to a previous study in dementia with Lewy body patients that demonstrated impaired orienting [Fuentes et al., 2010] in the absence of an alerting tone. The fact that two stimulatory modalities (auditory and visual) were used in this study compared to our study where both alerting and orienting elements were combined into one visual stimulus may explain differences between our study and that of Fuentes et al.

However despite the faster responses following the orienting cue, our study did not show any specific orienting effect on brain activation in either controls or LBD and only a small occipital activation in Alzheimer's disease, contradicting prior evidence which showed a subtle involvement of frontoparietal brain regions [Fan et al., 2005; Liu et al., 2013]. Indeed, the neutral cue had a slightly greater occipital activation than the directional cue, perhaps due to its more foveal presentation.

Examining the activation to target following the directional vs neutral cue, we found reduced occipital activity in Alzheimer's disease which was also almost significant for the control group (Fig. 7), suggesting that the cue, by drawing attention to the target location, leads to less visual search. On the other hand, in LBD, orienting led to increased activation of the insula on ROI analysis during target presentation. The insula is regarded to play a role in cognitive control activating task positive networks [Sidlauskaite et al., 2014] in response to salient stimuli [Downar et al., 2000; Downar et al., 2002]. One speculation is that insula activation in LBD might be a sign that the directional cue is increasing the salience of the upcoming target, although whether this is pathological or compensatory is unclear. Certainly inappropriate salience in the ventral attention network (in which the insula is a key node) has mechanistically been implicated in visual hallucination manifestation [Blanc et al., 2014; Shine et al., 2011] and it is notable that the insula appears to be particularly affected structurally in dementia with Lewy bodies, often early in the disease course [Zhong et al., 2014] highlighting the potential importance of this region in the pathophysiology of LBD.

### Executive Effect

We were able to demonstrate variation in RT with executive function demands in all groups. The error rate was consistently low in controls in both conditions, whereas it significantly increased in LBD and Alzheimer's disease when an incongruent target was presented. In terms of brain activity, all three groups had similar fronto‐occipito‐parietal activations to the targets, which were broadly similar to previous studies [Liu et al., 2013; Zheng et al., 2012], implying that LBD and Alzheimer's disease utilize the same distributed network of brain regions for attention as controls, with no areas of compensatory activity or deficit found. With increasing task difficulty, however, the dementia patients had greater increases in brain activity and slower RT compared with controls, which we would argue reflect compensations to maintain performance, given that dementia patients were able to maintain the same accuracy in easy and hard incongruent tasks.

These findings contrast with our a priori hypothesis of finding regional deficits, particularly in LBD. However, there is an increasing evidence base to suggest that large scale neocortical network alterations may be more pertinent to clinical and cognitive symptoms in LBD rather than specific regional nodes [Peraza et al., 2014; Taylor et al., 2013]. Connectivity analyses thus may be more suitable in LBD in delineating the basis of executive deficits [Peraza et al., 2015].

### Task‐Related Changes of the DMN In LBD and Alzheimer's Disease

We looked specifically at the role of the DMN during executive function by plotting its BOLD activation during different conditions. In agreement with a number of previous studies [Browndyke et al., 2013; Buckner et al., 2005; Rombouts et al., 2005], we found an absence of task related deactivation in Alzheimer's disease particularly of the posterior DMN during the incongruent task condition (Figs. 2, 5). Disruption of the DMN in Alzheimer's disease may be related to a distinct amyloid distribution [Buckner et al., 2005, Sperling, 2009]. Functionally, the lack of DMN deactivation during task performance could lead to failures in attentional allocation [Leech et al., 2011] and disintegration of executive functioning, resulting in less efficient decision making in Alzheimer's disease, as indicated by the increased error rates and RT.

Intriguingly the DMN demonstrated strong deactivation during both congruent and incongruent tasks in LBD. Previous studies have found mixed results regarding the DMN in dementia with Lewy bodies, although there is a tendency for it to demonstrate less impairment than in Alzheimer's disease [Franciotti et al., 2013; Kenny et al., 2012; Sauer et al., 2006]. Although the DMN showed, on average, an enhanced deactivation on target presentation in the present study, the gradient of deactivation in response to increasing target difficulty was of the same magnitude as in controls (Fig. 5). This might suggest that, whilst DMN activity is sufficiently modelled on task demand, there is an enhanced, albeit aberrant, attempt to switch cognitive resources from the DMN to task‐positive regions in LBD. Our finding that the LBD had greater deactivation for targets to which they either missed or responded incorrectly [Supporting Information Table S7] further implies that those targets requiring greater DMN deactivation were found to be more difficult to respond to. Deactivation to these levels, on a recurrent basis, even for low intensity cognitive tasks may have consequences in terms of fatigue and possibly alertness and cognitive fluctuations which typify LBD.

The strong deactivation, however was not accompanied by a comparable increase in activation of the fronto‐parietal networks with task difficulty. We hypothesize therefore that it is not a failure to deactivate the DMN that leads to inattention, which has been suggested to occur in young healthy controls [Weissman et al., 2006]; rather we speculate that inattention in LBD is likely to represent inefficiences in attentional networks and their dynamical synchronisation.

### Limitations and Future Directions

A number of limitations to our study need to be considered. Firstly, as we only used visual stimuli in our study, our findings are potentially confounded by the greater tendency for visuo‐perceptual deficits in LBD as evidenced with our angle discrimination task. Indeed, in response to our executive task, there was a tendency for the LBD patients to have more occipital activation compared with controls which could suggest inefficiencies in visual processing in this group. However this activation increase was relatively subtle and could have been driven by the longer attendence of the LBD patients to the target stimuli i.e. reflecting impaired top‐down attentional processing rather than aberrant bottom‐up visual processing. Further fMRI studies with tasks differentially weighted on attention and visual complexity may help address this question.

Another limitation was the effect of concurrent psychotropic medication. Cholinergic drugs are reported to improve attentional function in controls and dementia [Bentley et al., 2003; Broks et al., 1988; McKeith et al., 2000], mostly by modulating frontoparietal networks [Bentley et al., 2011; Bokde et al., 2009; Risacher et al., 2013]. All but five patients were on cholinesterase inhibitors, thus an effect of these cannot be ruled out. Dopaminergic treatment can also alter cognition and brain network function, albeit in a complex, dose dependent manner by differentially influencing orbital and dorsal frontostriatal loops [MacDonald et al., 2011] and possibly worsen attentional fluctuations [Molloy et al., 2006]. Notably we did not see any effects of these medications on our findings (unpublished data) but studies examining patients either not taking these medications or withdrawing them prior to imaging would help clarify the impact of these agents.

The LBD group had relatively few PDD subjects, since the recruited subjects in that group had more difficulty with motion in the scanner and performing the task, as well as some unrelated technical difficulties. This may have biased the LBD group toward a less motor predominant phenotype. DLB have also been reported to have more AD‐like amyloid pathology [Petrou et al., 2015], and it is possible that some level of amyloid deposition was present in the controls, leading to alterations in DMN deactivations. [Sperling et al., 2009] However, we did not measure amyloid levels, so we could not investigate the relationship between amyloid load and fMRI.

Depression can influence attention and cortical network activity, [Kertzman et al., 2010; Kikuchi et al., 2012] and it is notable that participants with LBD had greater Cornell depression scores. However, the average scores were well below cutoffs for clinical depression, and scores were relatively loaded to items which are likely to be higher in LBD (e.g. motor retardation, poor sleep etc.). Therefore depression is unlikely to have been a significant cofounder in the present study.

Finally, we did not examine dynamic interactions and connectivity between different regions in our present study, although our findings suggest a dynamic interplay between task‐positive and task‐negative regions in LBD and Alzheimer's disease during task performance. We also focussed our present analyses on cortical activity; however the pathophysiological role of key subcortical structures which provide corticopetal efferents to these neocortical networks such as the thalamus [Delli Pizzi et al., 2015] and Nucleus Basalis of Meynert [Gratwicke et al., 2015] are likely to be just as important. Further studies should explore the exact nature of the interaction between brain regions (cortical and subcortical) and their relationship to task performance by applying measures of functional and effective connectivity on a trial‐by‐trial basis; these analyses form part of our ongoing work.

## CONCLUSIONS

We found increased frontoparietal activation in LBD and Alzheimer's disease during attentional‐executive function in relation to task demand, with no regionally specific deficits nor recruitment of additional brain regions. Both the LBD and Alzheimer's disease patients had equally reduced performance compared to controls on the task. Despite these similarities; however, the dementia groups differed in the dynamic changes of the DMN. While in the LBD group we found a significantly increased DMN deactivation during target presentation, which was modulated by the task demand, the Alzheimer's disease group demonstrated limited task related deactivations in DMN regions. This has implications in the design of future clinical trials targeting attentional‐executive dysfunction in these disorders, suggesting that different therapeutic approaches may be needed in LBD compared with Alzheimer's to optimize outcomes.

## Supporting information

Supporting Information

Supporting Information

Supporting Information

Supporting Information

Supporting Information
