# Supplementary figures and images for: Neural correlates of attention‐executive dysfunction in lewy body dementia and Alzheimer's disease
Source: Hum Brain Mapp. 2015 Dec 26;37(3):1254–70. doi: 10.1002/hbm.23100 (PMC4784171; doi:10.1002/hbm.23100)

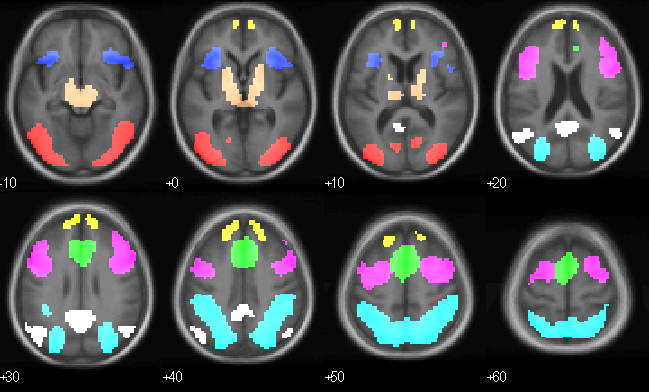

Supplement: Supplementary file 1 — Supporting Information [file HBM-37-1254-s001.tif]

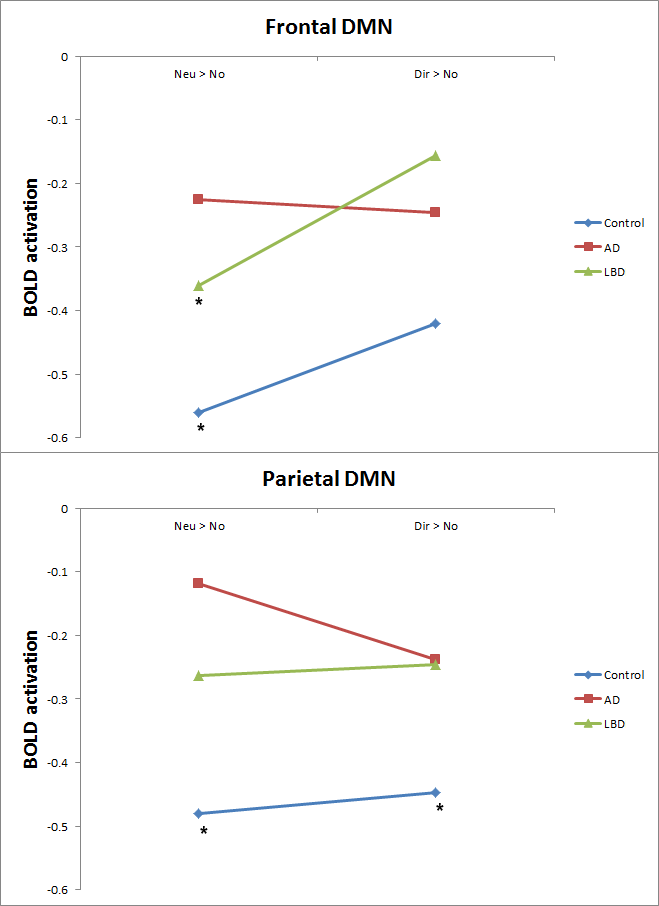

Supplement: Supplementary file 2 — Supporting Information [file HBM-37-1254-s002.tif]

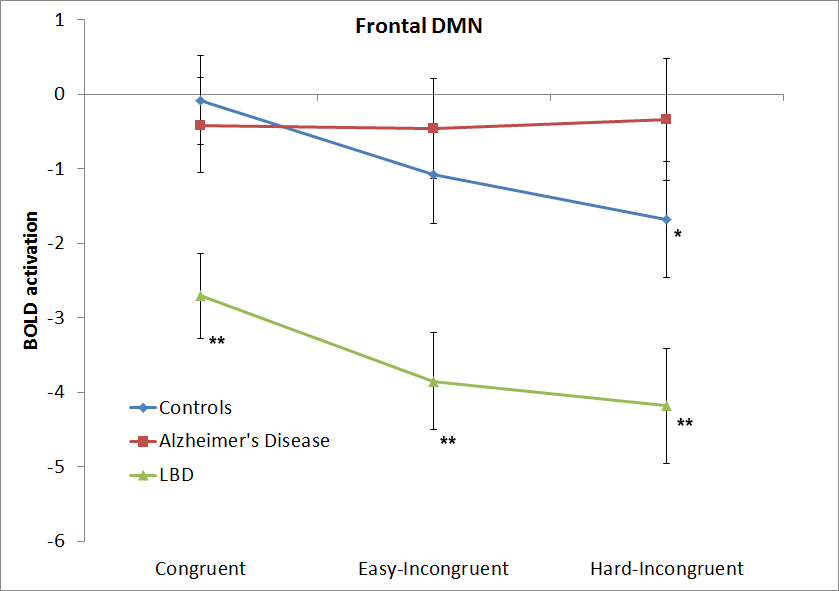

Supplement: Supplementary file 3 — Supporting Information [file HBM-37-1254-s003.tif]
