# Supplementary material for: Neural correlates of attention‐executive dysfunction in lewy body dementia and Alzheimer's disease
Source: Hum Brain Mapp. 2015 Dec 26;37(3):1254–70. doi: 10.1002/hbm.23100 (PMC4784171; doi:10.1002/hbm.23100)
Supplement: Supplementary file 4 — Supporting Information [file HBM-37-1254-s004.docx]

**Supplementary material**

**Figure S1.** **Regions of interest**.

These ROIs were defined from the incongruent vs congruent contrast in all participants (green – midline frontal, purple lateral frontal, dark blue: anterior insula, light blue: parietal, red: occipital, beige: thalamus/brainstem; white: parietal deactivation, yellow: frontal deactivation).

**Figure S2. ROI bold contrast to the neutral and directional cue vs no-cue.**

Within group significant deactivation indicated by * p<0.05

**Figure S3. BOLD contrast for the frontal DMN region during target presentation.**

Parietal DMN ROI analysis showing the BOLD contrast for the frontal DMN region for each group. Error bars are SE. Asterisk indicates within group contrasts (* p<0.05, ** p<0.01).

**Table S1. Demographics and clinical scores for dementia with Lewy bodies versus Parkinson’s disease with dementia.**

|  | Dementia with Lewy bodies (N=19) | Parkinson’s disease with dementia (N=13) |  |
| --- | --- | --- | --- |
| Age | 76.6 (6.3) | 72.6 (6.1) | T=1.8; p=0.08 |
| Sex M:F | 14:5 | 13:0 | P=0.064 (Fisher) |
| Duration cognitive | 3.55 (2.2) | 3.00 (1.96) |  |
| Cholinesterase Inhibitors | 17 (89%) | 10 (77%) | P=0.4 (Fisher) |
| Dopaminergic medication | 8 (42%) | 13 (100%) | P=0.001 (Fisher) |
| l-dopa equivalent dose (mg) | 350 (258) | 889 (423) | T=3.2, p=0.004 |
| +ve DAT scan | 10/11 (91 %) | - |  |
| UPDRS | 14.6 (5.2) | 25.5 (6.9) | T=5.1; p<0.001 |
| Cornell | 1.84 (1.6) | 4.62 (1.8) | T=4.6; p<0.001 |
| MMSE | 23.2 (4.3) | 23.6 (2.9) | T=0.3; p=0.8 |
| CAMCOG | 74.67 (15.34) | 79.7 (6.5) | T=1.1;p=0.3 |
| CAMCOG executive | 12.9 (4.9) | 13.9 (2.7) | T=0.6;p=0.5 |
| MAYO fluctuations | 1.95 (1.51) | 3.0 (0.85) | T=2.20;p=0.036 |
| MAYO cognitive | 2.16 (1.83) | 3.58 (1.73) | T=2.15;p=0.04 |
| CAF | 3.42 (4.0) | 7.00 (3.49) | T=2.55;p=0.016 |
| 1 day fluctuation | 2.71 (3.0) | 4.83 (2.98) | T=1.89;p=0.07 |
| NPI | 8.2 (5.5) | 21.1 (10.3) | T=4.6;p<0.001 |
| Visual Hallucinations | 11 (58%) | 11 (85%) | P=0.14 (Fisher) |
| Verbal fluency (FAS) | 21.0 (13.0) | 19.8 (10.6) | T=0.3;p=0.8 |
| Angle discrimination | 15.3 (5.3) | 18.4 (2.6) | T=1.9;p=0.07 |
| ANT trials responded % [range] | 96.3 [78.7 – 100] | 97.4 [88.0 – 100] | T=0.6;p=0.5 |
| % of trials with correct response | 85.6 [74.1 – 100] | 92.3 [80.1 – 100] | T=2.5;p=0.02 |
|  |  |  |  |

**Table S2. Reactions times, and differences in reaction time between conditions for dementia with Lewy bodies vs Parkinson’s disease with dementia.**

Executive contrast is the within subject difference in RT between Incongruent – Congruent targets. Conflict level is the difference between the easy and hard incongruent targets. Values in ms as mean (SD).

|  | Dementia with Lewy bodies (N=19) | Parkinson’s disease with dementia (N=13) | Dementia with Lewy bodies vs Parkinson’s disease with dementia t-test |
| --- | --- | --- | --- |
| Congruent | 1107 (234) | 1152 (307) | T=0.5;p=0.6 |
| Executive Contrast | 498 (239)## | 570 (133) ## | T=0.9;p=0.3 |
| Conflict level | 238 (259) ## | 338 (232) ## | T=1.1;p=0.3 |
| No Cue | 1358 (298) | 1451 (307) | T=0.9;p=0.4 |
| Alerting effect (no – neutral cue) | 8.1 (96) † | -0.3 (78) † | T=0.3;p=0.8 |
| Orienting effect (neutral – directional cue) | 61.3 (112) # | 73.1 (67) ## | T=0.3;p=0.7 |

Within group one sample t test for difference between cues / targets

# p < 0.05; ## p < 0.001 ; † not significant (p>0.15) . There were no sig differences dementia with Lewy bodies vs Parkinson’s disease with dementia (p > 0.25).

**Table S3.** Parkinson’s disease with dementia vs dementia with Lewy bodies ROI imaging analysis

| ROI | Dementia with Lewy bodies  BOLD activation | Parkinson’s disease with dementia BOLD activation | p (two sided) |
| --- | --- | --- | --- |
|  |  |  |  |
| **Congruent target** |  |  |  |
| Frontal Midline | 2.83 | 2.69 | 0.91 |
| Frontal Lateral | 1.72 | 0.99 | 0.46 |
| Insula | 2.58 | 1.77 | 0.50 |
| Parietal | 3.53 | 3.29 | 0.83 |
| Occipital | 5.65 | 6.22 | 0.64 |
| Thalamus / Brainstem | 2.31 | 2.67 | 0.73 |
| DMN Parietal | -2.98 | -4.33 | 0.24 |
| DMN Frontal | -2.56 | -2.86 | 0.80 |
|  |  |  |  |
| **Easy incongruent target** | |  |  |
| Frontal Midline | 4.57 | 5.29 | 0.63 |
| Frontal Lateral | 3.38 | 3.31 | 0.95 |
| Insula | 4.00 | 3.84 | 0.91 |
| Parietal | 5.78 | 6.51 | 0.58 |
| Occipital | 7.37 | 8.62 | 0.4 |
| Thalamus / Brainstem | 4.03 | 4.23 | 0.88 |
| DMN Parietal | -4.19 | -5.94 | 0.18 |
| DMN Frontal | -3.68 | -4.02 | 0.81 |
|  |  |  |  |
| **Hard incongruent target** | |  |  |
| Frontal Midline | 5.73 | 6.72 | 0.51 |
| Frontal Lateral | 4.64 | 4.84 | 0.87 |
| Insula | 4.82 | 5.85 | 0.54 |
| Parietal | 7.08 | 8.35 | 0.39 |
| Occipital | 9.37 | 10.06 | 0.7 |
| Thalamus / Brainstem | 5.05 | 5.24 | 0.88 |
| DMN Parietal | -4.97 | -7.19 | 0.16 |
| DMN Frontal | -3.64 | -4.73 | 0.49 |
|  |  |  |  |
| **Incongruent vs congruent** |  |  |  |
| Frontal Midline | 1.90 | 3.19 | 0.07 |
| Frontal Lateral | 2.07 | 3.02 | 0.15 |
| Insula | 1.61 | 3.08 | 0.1 |
| Parietal | 2.55 | 4.01 | 0.033 * |
| Occipital | 2.38 | 3.01 | 0.35 |
| Thalamus / Brainstem | 1.76 | 2.03 | 0.65 |
| DMN Parietal | -1.29 | -2.23 | 0.23 |
| DMN Frontal | -1.00 | -1.54 | 0.42 |
|  |  |  |  |
| **Easy vs hard incongruent** | |  |  |
| ROI |  |  |  |
| Frontal Midline | 1.16 | 1.43 | 0.76 |
| Frontal Lateral | 1.26 | 1.53 | 0.74 |
| Insula | 0.83 | 2.01 | 0.27 |
| Parietal | 1.31 | 1.85 | 0.57 |
| Occipital | 2.01 | 1.44 | 0.58 |
| Thalamus / Brainstem | 1.01 | 1.01 | 1 |
| DMN Parietal | -0.78 | -1.24 | 0.63 |
| DMN Frontal | 0.05 | -0.71 | 0.31 |
|  |  |  |  |
