# Supplementary material for: Neural correlates of attention‐executive dysfunction in lewy body dementia and Alzheimer's disease
Source: Hum Brain Mapp. 2015 Dec 26;37(3):1254–70. doi: 10.1002/hbm.23100 (PMC4784171; doi:10.1002/hbm.23100)
Supplement: Supplementary file 5 — Supporting Information [file HBM-37-1254-s005.docx]

**Supplementary material**

**Table S4. fMRI ROI comparison for contrasts between targets.** P values are two tailed. * = p < 0.05; ** p < 0.01 *** = <0.001

| **Incongruent vs congruent target** | | | | | | | | | |
| --- | --- | --- | --- | --- | --- | --- | --- | --- | --- |
|  | Controls | Alzheimer’s disease | LBD | Controls | Alzheimer’s disease | LBD | Controls vs Alzheimer’s disease | Controls vs LBD | Alzheimer’s disease vs LBD |
| ROI | BOLD | BOLD | BOLD | P value | P value | P value | P value | P value | P value |
| Frontal Midline | 2.09 | 3.24 | 2.55 | *** | *** | *** | 0.05 | 0.39 | 0.22 |
| Frontal Lateral | 2.43 | 3.20 | 2.54 | *** | *** | *** | 0.17 | 0.82 | 0.21 |
| Insula | 3.14 | 3.47 | 2.34 | *** | *** | *** | 0.65 | 0.23 | 0.11 |
| Parietal | 3.04 | 3.74 | 3.28 | *** | *** | *** | 0.23 | 0.65 | 0.4 |
| Occipital | 2.83 | 3.24 | 2.70 | *** | *** | *** | 0.48 | 0.79 | 0.33 |
| Thalamus / Brainstem | 1.75 | 2.45 | 1.90 | *** | *** | *** | 0.16 | 0.74 | 0.24 |
| DMN Parietal | -2.47 | -0.07 | -1.76 | *** | 0.88 | *** | *** | 0.23 | 0.008 ** |
| DMN Frontal | -1.30 | -0.07 | -1.27 | *** | 0.86 | *** | 0.032 * | 0.95 | 0.027 * |
| **Hard vs Easy Incongruent target** | | | | | | | | | |
|  | Controls | Alzheimer’s disease | LBD | Controls | Alzheimer’s disease | LBD | Controls vs Alzheimer’s disease | Controls vs LBD | Alzheimer’s disease vs LBD |
|  |  |  |  |  |  |  |  |  |  |
| Frontal Midline | -0.27 | 1.83 | 1.29 | 0.47 | *** | 0.004 ** | *** | 0.008 ** | 0.41 |
| Frontal Lateral | 0.00 | 1.84 | 1.40 | 0.99 | *** | *** | 0.001 ** | 0.01 * | 0.46 |
| Insula | -0.31 | 1.93 | 1.42 | 0.49 | 0.001 ** | 0.009 ** | 0.003 ** | 0.015 * | 0.51 |
| Parietal | 0.06 | 2.51 | 1.58 | 0.89 | *** | 0.001 ** | *** | 0.017 * | 0.19 |
| Occipital | 0.17 | 2.27 | 1.72 | 0.69 | *** | 0.001 ** | 0.003 ** | 0.022 * | 0.46 |
| Thalamus / Brainstem | -0.56 | 1.54 | 1.01 | 0.16 | 0.003 ** | 0.034 * | 0.002 ** | 0.012 * | 0.44 |
| DMN Parietal | -1.01 | 0.62 | -1.01 | 0.015 * | 0.24 | 0.038 * | 0.016 * | 0.99 | 0.024 * |
| DMN Frontal | -0.60 | 0.13 | -0.33 | 0.06 | 0.75 | 0.37 | 0.15 | 0.57 | 0.4 |

**Table S5 fMRI ROI response for individual targets compared to rest.** P values are two tailed. * = p < 0.05; ** p < 0.01 *** = <0.001

| **Congruent target** | | | | | | | | | |
| --- | --- | --- | --- | --- | --- | --- | --- | --- | --- |
|  | Controls  BOLD | Alzheimer’s disease  BOLD | LBD  BOLD | Controls  P value | Alzheimer’s disease  P value | LBD  P value | Controls vs Alzheimer’s disease  P value | Controls vs LBD  P value | Alzheimer’s disease v LBD  P value |
| Frontal Midline | 1.85 | 3.43 | 2.76 | 0.002 ** | *** | *** | 0.07 | 0.27 | 0.43 |
| Frontal Lateral | 0.97 | 1.94 | 1.35 | 0.06 | *** | 0.008 ** | 0.21 | 0.6 | 0.44 |
| Insula | 2.47 | 3.40 | 2.18 | *** | *** | *** | 0.31 | 0.74 | 0.18 |
| Parietal | 2.47 | 3.07 | 3.41 | *** | *** | *** | 0.47 | 0.23 | 0.68 |
| Occipital | 5.36 | 5.11 | 5.93 | *** | *** | *** | 0.78 | 0.51 | 0.36 |
| Thalamus / Brainstem | 3.44 | 4.04 | 2.49 | *** | *** | *** | 0.46 | 0.22 | 0.05 |
| DMN Parietal | 0.57 | 0.53 | -3.66 | 0.34 | 0.41 | *** | 0.96 | *** | *** |
| DMN Frontal | -0.08 | -0.42 | -2.71 | 0.89 | 0.53 | *** | 0.71 | 0.003 ** | 0.011 * |
|  |  |  |  |  |  |  |  |  |  |
| **Easy incongruent** | | | | | | | | | |
|  | Controls  BOLD | Alzheimer’s disease  BOLD | LBD  BOLD | Controls  P value | Alzheimer’s disease  P value | LBD  P value | Controls vs Alzheimer’s disease  P value | Controls vs LBD  P value | Alzheimer’s disease v LBD  P value |
| Frontal Midline | 4.07 | 6.20 | 4.93 | *** | *** | *** | 0.048 * | 0.41 | 0.24 |
| Frontal Lateral | 3.41 | 4.61 | 3.34 | *** | *** | *** | 0.16 | 0.94 | 0.14 |
| Insula | 5.76 | 6.17 | 3.92 | *** | *** | *** | 0.69 | 0.07 | 0.03 * |
| Parietal | 5.49 | 5.98 | 6.14 | *** | *** | *** | 0.6 | 0.48 | 0.87 |
| Occipital | 8.11 | 7.63 | 7.99 | *** | *** | *** | 0.65 | 0.91 | 0.73 |
| Thalamus / Brainstem | 5.47 | 5.93 | 4.13 | *** | *** | *** | 0.64 | 0.16 | 0.07 |
| DMN Parietal | -1.39 | 0.24 | -5.07 | 0.035 * | 0.73 | *** | 0.09 | *** | *** |
| DMN Frontal | -1.08 | -0.46 | -3.85 | 0.12 | 0.52 | *** | 0.54 | 0.006 ** | 0.001 ** |
|  |  |  |  |  |  |  |  |  |  |
| **Hard incongruent target** | | | | | | | | | |
|  | Controls  BOLD | Alzheimer’s disease  BOLD | LBD  BOLD | Controls  P value | Alzheimer’s disease  P value | LBD  P value | Controls vs Alzheimer’s disease  P value | Controls vs LBD  P value | Alzheimer’s disease v LBD  P value |
| Frontal Midline | 3.80 | 8.03 | 6.23 | *** | *** | *** | *** | 0.026 * | 0.11 |
| Frontal Lateral | 3.40 | 6.45 | 4.74 | *** | *** | *** | 0.001 ** | 0.14 | 0.07 |
| Insula | 5.45 | 8.10 | 5.34 | *** | *** | *** | 0.033 * | 0.92 | 0.026 * |
| Parietal | 5.54 | 8.50 | 7.72 | *** | *** | *** | 0.007 ** | 0.039 * | 0.47 |
| Occipital | 8.28 | 9.90 | 9.72 | *** | *** | *** | 0.22 | 0.26 | 0.89 |
| Thalamus / Brainstem | 4.91 | 7.47 | 5.15 | *** | *** | *** | 0.009 ** | 0.8 | 0.017 * |
| DMN Parietal | -2.40 | 0.85 | -6.08 | 0.003 ** | 0.3 | *** | 0.005 ** | 0.001 ** | *** |
| DMN Frontal | -1.69 | -0.34 | -4.18 | 0.036 * | 0.69 | *** | 0.24 | 0.028 * | 0.001 ** |

**Table S6** fMRI Activation in ROI on targets due to different cues**.** P values are two tailed. * = p < 0.05; ** p < 0.01 *** = <0.001

| **Targets following No Cue** | | | | | | | | | |
| --- | --- | --- | --- | --- | --- | --- | --- | --- | --- |
|  | Controls BOLD | Alzheimer’s disease BOLD | LBD  BOLD | Controls  P value | Alzheimer’s disease  P value | LBD  P value | Controls vs Alzheimer’s disease P value | Controls vs LBD  P value | Alzheimer’s disease v LBD P value |
| Frontal Midline | 3.65 | 7.00 | 2.63 | 0.002 ** | *** | 0.007 ** | 0.036 * | 0.49 | 0.003 ** |
| Frontal Lateral | 3.29 | 5.53 | 1.41 | *** | *** | 0.07 | 0.08 | 0.12 | *** |
| Insula | 4.3 | 3.59 | 1.67 | *** | 0.003 ** | 0.1 | 0.67 | 0.1 | 0.22 |
| Parietal | 4.56 | 7.38 | 3.18 | *** | *** | *** | 0.06 | 0.33 | 0.003 ** |
| Occipital | 6.88 | 8.18 | 5.47 | *** | *** | *** | 0.44 | 0.37 | 0.08 |
| Thalamus / Brainstem | 4.42 | 5.65 | 2.62 | *** | <0.001 ** | 0.003 ** | 0.38 | 0.17 | 0.021 * |
| DMN Parietal | -2.9 | 1.99 | -5.11 | 0.032 * | 0.12 | *** | 0.01 ** | 0.2 | *** |
| DMN Frontal | -1.92 | 0.69 | -4.98 | 0.17 | 0.61 | *** | 0.18 | 0.09 | 0.002 ** |
|  |  |  |  |  |  |  |  |  |  |
| **Targets following Neutral Cue** | | | | | | | | | |
|  | Controls BOLD | Alzheimer’s disease BOLD | LBD BOLD | Controls  P value | Alzheimer’s disease  P value | LBD  P value | Controls vs Alzheimer’s disease P value | Controls vs LBD  P value | Alzheimer’s disease v LBD P value |
| Frontal Midline | 1.84 | 3.98 | 4.38 | 0.019 * | *** | *** | 0.07 | 0.019 * | 0.73 |
| Frontal Lateral | 1.59 | 2.49 | 2.8 | 0.024 * | 0.002 ** | *** | 0.39 | 0.2 | 0.76 |
| Insula | 1.84 | 2.82 | 3.1 | 0.031 * | 0.004 ** | *** | 0.44 | 0.28 | 0.82 |
| Parietal | 3.17 | 5.07 | 5.26 | *** | *** | *** | 0.14 | 0.08 | 0.88 |
| Occipital | 6.63 | 6.92 | 6.59 | *** | *** | *** | 0.81 | 0.97 | 0.78 |
| Thalamus / Brainstem | 3.59 | 5.09 | 3.08 | *** | *** | *** | 0.14 | 0.58 | 0.043 * |
| DMN Parietal | -0.57 | 0.16 | -4.28 | 0.49 | 0.86 | *** | 0.56 | 0.002 ** | *** |
| DMN Frontal | -0.75 | -2.38 | -4.06 | 0.38 | 0.015 * | *** | 0.2 | 0.005 ** | 0.18 |
|  |  |  |  |  |  |  |  |  |  |
| **Targets following Directional Cue** | | | | | | | | | |
|  | Controls  BOLD | Alzheimer’s disease  BOLD | LBD  BOLD | Controls  P value | Alzheimer’s disease  P value | LBD  P value | Controls vs Alzheimer’s disease  P value | Controls vs LBD  P value | Alzheimer’s disease v LBD  P value |
| Frontal Midline | 1.79 | 3.69 | 4.73 | 0.021 * | *** | *** | 0.09 | 0.006 ** | 0.35 |
| Frontal Lateral | 1.57 | 2.26 | 2.96 | 0.016 * | 0.002 ** | *** | 0.46 | 0.12 | 0.45 |
| Insula | 1.93 | 2.22 | 3.83 | 0.045 * | 0.034 * | *** | 0.84 | 0.15 | 0.24 |
| Parietal | 2.72 | 4.43 | 5.38 | 0.002 ** | *** | *** | 0.17 | 0.022 * | 0.43 |
| Occipital | 5.81 | 6.11 | 6.96 | *** | *** | *** | 0.79 | 0.27 | 0.43 |
| Thalamus / Brainstem | 3.61 | 4.4 | 3.54 | *** | *** | *** | 0.42 | 0.94 | 0.36 |
| DMN Parietal | -1.06 | -0.14 | -3.72 | 0.2 | 0.88 | *** | 0.44 | 0.02 * | 0.003 ** |
| DMN Frontal | -0.87 | -2.56 | -3.57 | 0.28 | 0.005 ** | *** | 0.16 | 0.017 * | 0.38 |

**Table S7.** Invalid vs validly responded to target contrast in the ROIs. P values are two sided. * = p<0.05; ** p<0.01. There are 10 Alzheimer’s disease, 14 dementia with Lewy bodies and 7 Parkinson’s disease with dementia included in the analysis

|  | Alzheimer’s disease | LBD | Alzheimer’s disease | LBD | Alzheimer’s disease vs LBD |
| --- | --- | --- | --- | --- | --- |
|  | BOLD beta | BOLD beta | P value | P value | P value |
| Frontal Midline | 2.14 | 3.18 | 0.05 | <0.001 ** | 0.39 |
| Frontal Lateral | 1.10 | 2.10 | 0.38 | 0.002 ** | 0.47 |
| Insula | 4.44 | 2.08 | 0.001 ** | 0.003 ** | 0.11 |
| Parietal | 2.88 | 3.91 | 0.08 | <0.001 ** | 0.56 |
| Occipital | 5.10 | 5.80 | 0.001 ** | <0.001 ** | 0.66 |
| Thalamus / Brainstem | 3.00 | 2.78 | 0.011 * | <0.001 ** | 0.86 |
| DMN Parietal | -1.10 | -3.58 | 0.51 | <0.001 ** | 0.19 |
| DMN Frontal | -2.43 | -3.22 | 0.15 | <0.001 ** | 0.67 |
